# Preliminary Results of Bone Regeneration in Oromaxillomandibular Surgery Using Synthetic Granular Graft

**DOI:** 10.1155/2018/8503427

**Published:** 2018-11-05

**Authors:** Noemi Mazzone, E. Mici, A. Calvo, M. Runci, S. Crimi, F. Lauritano, E. Belli

**Affiliations:** ^1^Unit of Maxillo-Facial Surgery, University of Rome “Sapienza”, Azienda Ospedaliera Universitaria “Sant'Andrea”, Rome, Italy; ^2^Unit of Maxillo-Facial Surgery, University Messina, Azienda Ospedaliera Universitaria “G. Martino”, Messina, Italy; ^3^Department of Biomedical and Dental Sciences and Morphological and Functional Imaging, University of Messina, 98100 Messina, Italy

## Abstract

Traumatic, neoplastic, inflammatory, or infective dental removal promotes a gradual resorption process of bone which leads to a “nonuse” atrophy of the alveolar ridges. Many techniques allows restoring an appropriate bone thickness, but nowadays the attention is focused on the use of natural or synthetic grafts. Numerous studies have been conducted to develop and test new synthetic materials. In this article, the authors report their experience using a synthetic bone substitute in combination with Platelet Rich Fibrin (PRF). This technique was applied in different zones of the maxillomandibular district. The procedure showed a very satisfying bone regeneration without important complications.

## 1. Introduction

Traumatic, inflammatory, or infective dental removal lead to an atrophy of the alveolar ridges. This is in accordance with the postulate that Julius Wolff made in the mid-1800s: the pressure and the loading force of the teeth are fundamental for the maintenance of the quality and quantity of the alveolar bone [1]. After the tooth loss, the alveolar process reduces in a centripetal way, before in width and then in height. This process is completed within 3-4 months. The resorption process can be faster and more severe in patients affected by systemic disease or local inflammation [2]. Also in the maxillofacial district, traumas, oncological disease, and more frequently cysts of the jaws lead to bone or teeth loss. Many techniques are used nowadays for filling the defects caused by those affections. Obviously, a dental rehabilitation is needed in most cases. An adequate bone regeneration is the first step to a successful implant rehabilitation [3], and a certain residual amount of bone is required for that [4]. The developing of new technologies and of the regenerative medicine has led to the improvement of the success rates in bone grafting [5, 6]. Bone grafts are classified into autogenous grafts, allografts, xenografts, and synthetic grafts, depending on the donor tissue and the material [7]. Autogenous bone is taken from an intra- or extra-oral donor site. It can be splitted or left in blocks and then inserted into a receiving site of the same patient. Because of its osteogenic and osteoconductive properties, it is considered the gold standard for bone augmentation. Thus, it has higher costs, potential graft resorption, and morbidity of the donor site and needs a second surgery time [8]. Due to those inconveniences, to date, several studies were conducted to develop and test new synthetic materials. Those replacement materials consist of one or more components: an osteoconductive matrix, which supports the growth of new bone, and osteoinductive proteins, which sustain mitogenesis of undifferentiated cells, and osteogenic cells (osteoblasts or osteoblast precursors), which are capable of forming bone in the proper environment [9]. Those methods are used for the production of synthetic grafts that can be produced in big quantities and applied without causing adverse reactions [10]. However, they remain at risk for resorption and infection due to donor tissue [11]. Jo et al. have compared the bovine mineral deproteinized bone (Bio Oss ®) with the synthetic hydroxyapatite (HA), used individually and after coverage with fibronectin to evaluate the adhesion of bone marrow stromal cells on both materials in vitro, demonstrating the superiority of the synthetic hydroxyapatite in providing a favorable environment for cell attachment [12]. Kim et al. reported excellent advantages of synthetic substitute in terms of availability and production. Effectively, there is no risk of disease transmission and immune-relate affections, obtaining satisfying physical properties and plastic resorption degree [13]. This grafts can be inserted with an “onlay” or with an “inlay” technique. In the onlay technique the bone resorption can be not predicted and sometimes can arrive till 50% of the graft. The inlay technique has shown better results [3, 14]. In this retrospective study, we report our experience of bone regeneration using synthetic bone substitute.

## 2. Materials and Methods

**Figure 1:**
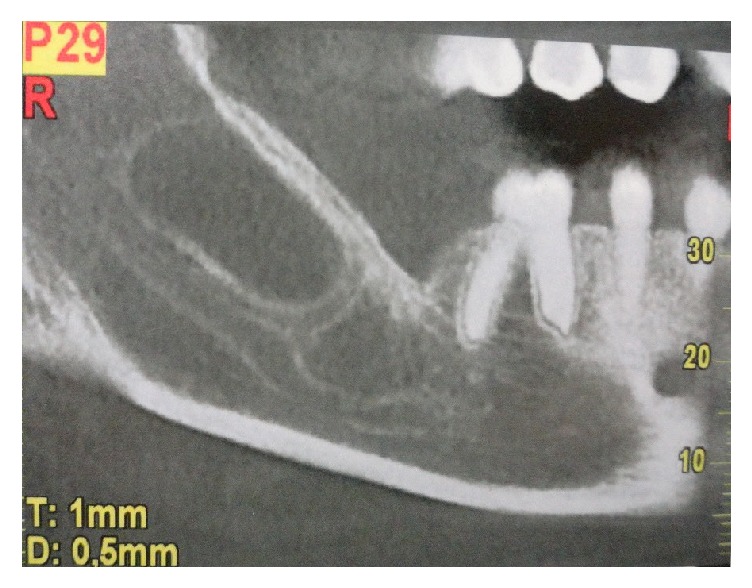
Orthopanoramic view that shows a right mandibular cystic lesion.

**Figure 2:**
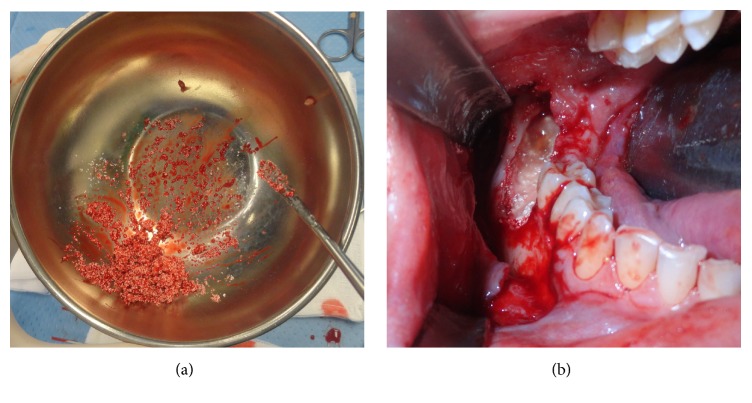
(a) The granular bone is mixed with PRF. (b) Intraoperative view of the filled bone defect covered with PRF membranes.

Between April 2015 and September 2016, 15 patients were evaluated (mean age 45 years), in the Maxillofacial Surgery Department of Sant'Andrea Hospital, Rome. Four patients were women and eleven were men. Of those patients, 11 were affected by jaws cysts, 2 had trauma outcomes, and 2 had jaw atrophy outcomes. Furthermore, 9 cases presented with mandibular involvement, 5 cases with maxillary involvement, and 1 patient presented zygomatic involvement (Table 1). Pregnant patients, heavy smokers, low mouth hygiene, and oncologic patients were excluded from this treatment. Preoperatively photographs were taken and all patients underwent Orthopanoramic X-Ray and Dentalscan CT (Figure 1). Patients were well informed about type of material and surgery and a written informed consent was obtained. The used graft, ReOss® (Intra-Lock International, Inc.), is a biphasic resorbable biomaterial composed by 50% of bioceramic synthetic hydroxyapatite and 50% of biodegradable polymer of poly (lactic-co-glycolic). This, was used in combination with Platelet Rich Fibrin (PRF). This is a Platelet Rich Plasma (PRP) II generation derivate in which, however, the platelet degranulation has generated cytokines and growth factors storage within the fibrin clot. They promote neoangiogenesis and facilitate the healing and graft integration thank to their rich content of growth factors like basic fibroblast growth factor (bFGF), vascular endothelial growth factor (VEGF), platelet-derived growth factor (PDGF), and platelet microparticles (PMPs) [15]. The used graft was a synthetic bone substitute, a biomaterial composed by 50% of resorbable biphasic bioceramics of HA and 50% of biodegradable polymer of polylactic acid-co-glycolic acid (PLGA). This biomaterial was combined intraoperatively with platelet reach fibrin (PRF). The PRF was used in pieces and in membranes following the Choukroun technique [16]. Patients were treated surgically in general or local anesthesia depending on the disease entity. The material quantity to be grafted was previously established and eventually corrected intraoperatively. The PRF preparation was performed during surgery by patient's venous blood sample. The blood was collected in 10 milliliter vacuum tubes. The tubes were inserted in a centrifuge and centrifugated in 3000 rotation/minute for 10 minutes. Then the upper part of the formatted clot was taken and it was compressed in order to create membranes. Bone defects were filled with a combination of synthetic bone and PRF. Granular bone was mixed with PRF to form a homogeneous compound (Figure 2(a)). The defect was filled with this compound and then covered with PRF membranes (Figure 2(b)). In the postoperative time, antibiotic therapy and analgesic therapy were administered for 6 and 3 days, respectively. The patients were discharged medially 2 days after surgery. Soft diet and accurate oral hygiene was indicated. Follow-up was performed at 3, 6, and 12 days after surgery.

## 3. Results

**Figure 3:**
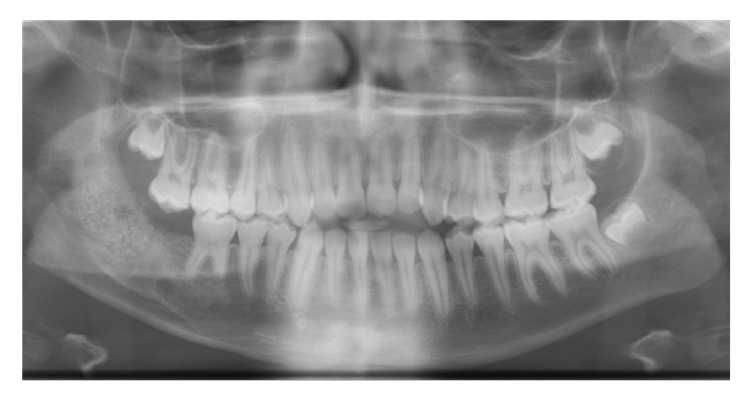
Orthopanoramic postoperative view that shows a satisfying healing of the bone defect.

**Figure 4:**
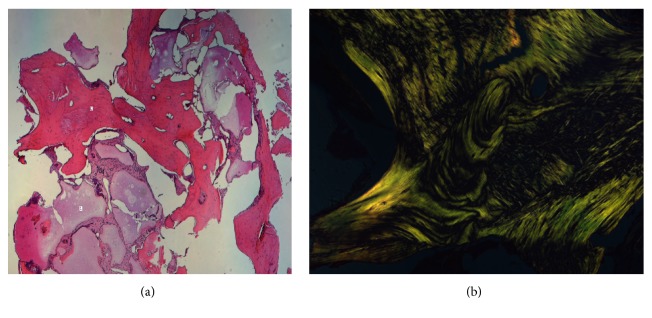
(a) Microscopic view X 25 shows lamellar bone, osteoid tissue mixed to fibrous tissue, and liquid. O (bone); L (liquid). (b) Microscopic view with polarized light X 100, normal mature bone tissue with lamellar collagenous tissue in the osteon.

In this study were evaluated 15 patients (age ranged between 12 and 71 years). After surgery, all patients underwent clinical and radiological follow-up. Surgical site infection occurred in 1 patient and in the 15th postoperative day the graft was removed surgically and antibiotic therapy was administered. In 4 cases (26,5%) surgical wound dehiscence occurred but was resolved in 3-4 weeks, and in any case the bone regeneration process was not compromised. The mean follow-up was 8.9 months (range: 25 days up to 17 months). Patients underwent Orthopantomography X-Ray and Dentalscan CT at 3 and 6 months after surgery (Figure 3). In addition, a histopathological exam was performed after 6 months to evaluate the status and quantity of bone replacement and quality of the new formed one (Figures 4(a) and 4(b)). In general, all patients showed a satisfactory engraftment, with effective native bone regeneration at 3 months after surgery.

## 4. Discussion

Malformation, traumatic, neoplastic, infectious, or inflammatory conditions can bring to bone loss or resorption. That can lead to a deprivation of the alveolar bone of the mechanical stimulus and load that each tooth exerts on the corresponding ridge portion. An adequate bone regeneration is required before a teeth rehabilitation. Hirsch et al. have attested a fairly low success rate of implant positioning in atrophic ridges without a preprosthetic surgery. It varies between 27 and 37%, highlighting the concept of adequate bone thickness [17, 18]. Bone grafts are classified into autogenous bone grafts, allografts, xenografts, and synthetic bone grafts depending on donor tissues and materials. Among different possibilities for bone regeneration, the autologous bone ensures the best results for the immediate engraftment, the excellent consolidation, and continued rapid bone formation, but its use is mainly limited by the material scarcity, morbidity rates and costs [19, 20]. For solving those problems, to date, several synthetic materials were developed. Those replacement materials consist of one or more components: an osteoconductive matrix, which supports the growth of new bone; osteoinductive proteins, which sustain mitogenesis of undifferentiated cells; and osteogenic cells (osteoblasts or osteoblast precursors), which are capable of forming bone in the proper environment. The synthetic bone showed ability to induce bone formation comparable to the heterologous grafts, without presenting the related risks [21]. Among several alternatives, the alloplastic materials most widely used are derived from the bioceramics of calcium phosphate, namely tricalcium phosphate and hydroxyapatite (HA). In the last 20 years the alloplastic materials find application in dental and maxillofacial surgery, plastic surgery, and orthopedic surgery This has several forms such as nonabsorbable (in turn porous or dense) and resorbable forms. The nonabsorbable material expresses good bone conductivity. Absorbable materials should be used when graft replacement by natural bone is needed. The slow resorption of the resorbable material allows operating as a mineral reservoir and as a scaffold for substitution with native bone [22, 23]. Recently, PRF is used in combination with alloplastic materials. It has angiogenic, proliferative, and differentiation effects due to the transforming growth factor-beta (TGF-beta) and the Platelet-derived growth factor (PDGF) that are present in high concentrations. In addition, it promotes wound healing, growth and bone maturation. The combination with PRF stabilizes bone grafting and promotes hemostasis and better handling of the graft material [24]. The PRF is a revolutionary step in the use of platelet gel derived. Indeed, platelets and leukocytes play an important role in the activity of this biomaterial, but the fibrin matrix that supports them is responsible for the therapeutic properties. Cytokines are immediately consumed and degraded during the healing process. Harmony between the cytokines and the fibrin matrix is by far the most important factor for this effect [25]. A recent study has shown that the PRF membranes are characterized by a slow and gradual release of growth factors for at least one week and up to 28 days after the application. It means that such membranes stimulate the microenvironment in which they are grafted for a significant period of time during the healing of the surgical wound [26]. Although various materials are available for graft regenerative surgery, the ideal material for such procedures has not been clearly identified yet. Pappalardo et al. have attributed most osteoconductive properties to demineralized freeze-dried bone (DFDBA) and Lyophilized bovine bone (Bio-Oss) compared to PLGA/HA combination [27]. Kim et al. have come to opposite conclusions by studying the regeneration with Bio Oss ® and PLGA/HA of bone defects in animal models [13]. There are numerous studies on animal models, with sometimes conflicting results, but a few studies validated on human models on the PLGA/HA combination. There is only one recent study in the literature that has compared the bovine lyophilized bone with PLGA/HA thus allowing a more accurate assessment of the actual osteoconductive capacity [28]. The thickness and bone radiodensity were slightly higher in the group treated with the bovine bone, probably to a greater rate of resorption and less persistence of PLGA/HA. The authors consider that these characteristics reduce healing time and therefore reduced time between bone graft and implant placement.

## 5. Conclusions

In this study, we assessed bone regeneration using a new biomaterial (PLGA/HA). There is only one previous known study in the literature. The regenerated bone was evaluated by radiological examinations and histological examination. Within the limits of this study, these results support the ability of this material to regenerate bone in sufficient quantity and quality for subsequent implants rehabilitation. Further studies needed to evaluate complications and their management, in order to further reduce their incidence.

## Figures and Tables

**Table 1:** Diseases and bone involvement.

	Cysts	Trauma	Atrophy
Mandibular	6	2	1*∗*
Maxillary	3	1	1*∗*
Zygomatic	1	/	/

## Data Availability

The data used to support the findings of this study are available from the corresponding author upon request.
